# Microsatellites and SNPs linkage analysis in a Sardinian genetic isolate confirms several essential hypertension loci previously identified in different populations

**DOI:** 10.1186/1471-2350-10-81

**Published:** 2009-08-28

**Authors:** Evelina Mocci, Maria P Concas, Manuela Fanciulli, Nicola Pirastu, Mauro Adamo, Valentina Cabras, Cristina Fraumene, Ivana Persico, Alessandro Sassu, Andrea Picciau, Dionigio A Prodi, Donatella Serra, Ginevra Biino, Mario Pirastu, Andrea Angius

**Affiliations:** 1 Shardna Life Sciences, Pula (Cagliari), Italy; 2 CNR, Department of Life Sciences, Institute of Population Genetics, Li Punti (Sassari), Italy

## Abstract

**Background:**

A multiplicity of study designs such as gene candidate analysis, genome wide search (GWS) and, recently, whole genome association studies have been employed for the identification of the genetic components of essential hypertension (EH). Several genome-wide linkage studies of EH and blood pressure-related phenotypes demonstrate that there is no single locus with a major effect while several genomic regions likely to contain EH-susceptibility loci were validated by multiple studies.

**Methods:**

We carried out the clinical assessment of the entire adult population in a Sardinian village (Talana) and we analyzed 16 selected families with 62 hypertensive subjects out of 267 individuals. We carried out a double GWS using a set of 902 uniformly spaced microsatellites and a high-density SNPs map on the same group of families.

**Results:**

Three loci were identified by both microsatellites and SNP scans and the obtained linkage results showed a remarkable degree of similarity. These loci were identified on chromosome 2q24, 11q23.1–25 and 13q14.11–21.33. Further support to these findings is their broad description present in literature associated to EH or related phenotypes. Bioinformatic investigation of these loci shows several potential EH candidate genes, several of whom already associated to blood pressure regulation pathways.

**Conclusion:**

Our search for major susceptibility EH genetic factors evidences that EH in the genetic isolate of Talana is due to the contribution of several genes contained in loci identified and replicated by earlier findings in different human populations.

## Background

Essential hypertension (EH [MIM 145500]), defined as chronically elevated blood pressure (BP) occurring in the absence of other predisposing conditions, has a worldwide prevalence of nearly 27% [1,2]. It is a major risk factor for stroke, heart disease and end-stage renal disease [3]. Twin studies support the existence of genetic factors and studies on the genetic basis of susceptibility to hypertension have estimated that the heritability of blood pressure (BP) levels is around 30%–35% [4]. EH is considered a multifactorial disorder caused by multiple genetic variants, gene-gene interactions and environmental factors [5].

Rare syndromic forms of hypertension show Mendelian inheritance and some variants related to several of these disorders were identified through positional cloning and candidate-gene analyses [6]. Mutations underlying monogenic forms of hypertension are rare in the general population. In most cases the risk to developing EH might be related to multiple variants, each with small and intermediate effect and/or with an additive effect on pathways related to EH [7,8].

The study of the genetic components of hypertension is carried out by different approaches such as gene candidate analysis, genome wide search (GWS) and, recently, by whole genome association studies [9,10]. Several genome-wide linkage studies of EH and BP-related phenotypes using populations of different origin demonstrate that there is no single genomic region with a large effect on BP or EH [11,12]. However, several genomic regions, such as 1q, 2p, 3p, 6q, 7q, 12q, 15q, 17q, 18q, and 19p, were validated by multiple studies and are likely to contain EH-susceptibility loci [5,13]. These loci may contain hundreds of genes and linkage studies using microsatellite markers are not able to restrict these regions. Until recently, the majority of hypertension GWS were performed using low-density maps of STR markers with a spacing of about 5–10 cM across the genome.

The improvement in high-throughput technologies for single nucleotide polymorphisms (SNPs) genotyping has opened up a rapid and economical way of whole genome analysis. International databases containing millions SNPs provide a highly dense map across the human genome, and it is possible to achieve adequate power for investigating genome variants associated to complex diseases such as hypertension. Previous studies demonstrated that a highly dense map of SNP markers can increase the power to detect linkage and consequently identify a disease locus more precisely [14] than would be with low-density microsatellites maps.

To reduce genetic complexity, our group chose to study Sardinian isolated populations where it is expected to find a simplification of complex disease models [15,16]. As a matter of fact, the probability of finding susceptibility genes is higher because people share a common environment and an age-stratified uniform lifestyle minimizing non-genetic variability and maximizing genetic effect.

In this study, we report a genome-wide linkage analysis of families affected by essential hypertension in a genetic isolate called Talana located in the Ogliastra region in Central-Eastern Sardinia. Mitochondrial analysis traces this village original population back to the Neolithic era and reveals one of the most genetically homogenous populations so far described [17]. We chose Talana for its peculiar genetic features such as increased background linkage disequilibrium [18] due to its demographic history that has led to genetic drift and founder effects. We evaluate LD parameters and effective population structure using a dense whole genome SNPs map that evidence high values of LD parameters and the presence of LD variability across the genome [19]. There are potential advantages in the choice of these kind of populations because of reduced environmental complexity and a probably reduced number of disease alleles. Some of the loci are expected to have stronger genetic effects on the disease/trait under analysis.

Here, we discuss the findings of whole genome analysis in several families with patients affected by essential hypertension living in Talana. Our genome-wide linkage analysis were obtained utilizing an intermediately-dense STRs set and a highly-dense SNPs map.

## Methods

### Population features

The Central-Eastern area of Sardinia called Ogliastra is characterized by the presence of several distinct villages with different history, immigration events and population size [20]. Centenarian cultural isolation and conservatism characterized the history of these communities. The relevant features of this geographic area are similar environmental conditions, high endogamy, low immigration and remote origin. This region also appears genetically differentiated from neighboring areas [19]. Our study has focused on one of the most isolated of these villages called Talana. During the past, this community (1127 residents), lying in a mountainous zone, maintained few contacts and exchanged few marriages with other villages in the same geographical area. Genetic analyses of Y chromosome and mitochondrial DNA [17] show that more than 80% of the present-day population descend from about twenty founders. This population has a higher level of endogamy than what is found in the whole Ogliastra: 92% in Talana compared to the regional 80%. Extensive historical and archival data allow an accurate reconstruction of the genealogy of each extant individual. These data are collected in a structured and comprehensive genealogy database that was used to cluster individuals in pedigrees that connect up to 16 generations [21]. We used patient list as input and utilized an application designed to find in the database all the ancestors related to any member on the input list.

### Clinical assessment

People living in the village were invited to take part in the study by invitations sent by mail to every family and through public announcements. All the people participating in the study were volunteers. The personal data were encrypted and kept separated from genetic and data. The scientific content of the project was extensively explained to all individuals participating in the study that signed informed consent forms in accordance with the Helsinki Declaration . The study was submitted and approved to the Italian Ministry of University and Research (MIUR) following the current Italian legislation.

Respondents underwent blood tests, anthropometric and blood pressure measurements. Each volunteer was interviewed by a health worker who collected socio demographic information, clinical and family history, medication history and information about important risk factors for EH: living habits, such as smoking, alcohol consumption and physical activity.

BP was measured in both arms at initial assessment, at the end of the 40 minutes interview and the arm with the highest pressure was used consequently. After 2 or more measurements, done according to the Joint National Committee on Prevention, Detection, Evaluation, and Treatment of High Blood Pressure [22], if the person had a systolic blood pressure (SBP) ≥ 140 mmHg or a diastolic blood pressure (DBP) ≥ 90 mmHg, he/she was then invited to undergo new measurement sessions in the next few weeks. BP measurements were obtained from sitting subjects and with at least two minutes interval between each cuff inflation. Standard mercury sphygmomanometers (Miniatur 300 B, Speidel & Keller) were used, and one of 4 cuff sizes (pediatric, regular adult, large, or tight) was chosen on the basis of the participant's arm circumference. The auscultation method was used: Korotkoff phase I and phase V sounds were used in order to determine SBP and DBP levels.

Biochemical analyses (24 serologic and 22 hemogram parameters) were done in our central laboratory in Perdasdefogu. Hematology parameters were evaluated using the Coulter AcT 5 autoloader hematology analyzer from Beckman Coulter (Fullerton, CA, USA). We directly measured CBC parameters (white blood cell count, red blood cell count, hemoglobin, mean corpuscular volume and platelets) and calculated coefficients following the manufacturer's specifications. Basic chemistries, electrolytes, enzymes, full lipid profile and derived tests were performed by using the VITROS dry-slide technology an automated Vitros 250 Chemistry System (Orthoclinical Diagnostics, Johnson & Johnson Gateway SM, US). We analyzed the following serum parameters: fasting glucose, creatinine, sodium, potassium, calcium, phosphorus, chloride, magnesium, uricaemia, GOT/AST, GPT/ALT, bilirubin, protein, albumin, cholinesterase, alkaline phosphates, cholesterol and triglycerides. LDL-C levels was determined by the Friedewald formula: LDL-C = TC - (HDL-C + TG/5).

For anthropometric measurements subjects were examined wearing only their underwear. Weight was determined on a portable electronic scale to the nearest 0.1 kg and height was measured to the nearest 0.5 cm with a stadiometer. Subjects were without shoes, standing upright with the head in the Frank-fort plane for height measurements. The body mass index (BMI) in kg/m^2^ was calculated from weight divided by height squared.

Physicians entered all collected data on PCs using an electronic medical record based on the structured interview drawn up by the study group. Such an electronic medical record is made of several modules allowing consultation of a pharmacological archive, according to the ATC classification, in order to correctly enter medicines taken by patients and consultation of the ICD-IX for the correct pathological classification. The common purpose of every module is to obtain standardized information.

Hypertension or high BP was defined as mean SBP ≥ 140 mmHg, mean DBP ≥ 90 mmHg or current treatment for hypertension, verifying the patient information on the slips that the participants were invited to bring with them to the interview. Awareness of hypertension reflects any prior diagnosis of hypertension or high BP by a health professional among the participants defined as having high BP. Control of hypertension was defined as pharmacological treatment of hypertension associated with SBP < 140 mm Hg and DBP < 90 mm Hg.

Our study focused on essential hypertension and, for this reason, we excluded all diabetic individuals, patients with renal diseases and people with certain or possible secondary hypertension on the bases of the anamnesis and the information provided by the local doctor that follow the large part of the patients during the recent years. In particular, for the latter case we excluded obese people, patients with insufficient or negative response to therapeutic drugs or characterized by a rapid and marked increase in blood pressure.

For the linkage study, we selected 99 severe hypertensive subjects based on these criteria: i) age at onset ≤ 65 yrs; ii) BMI ≤ 35; iii) PAD ≥ 95 mmHg or antihypertensive therapy; iv) exclusion of diabetes; v) exclusion of secondary hypertension; vi) exclusion of individuals with high consumption of smoke and alcohol.

The choice of a rather elevated BMI cut off point was determined by the average BMI in Talana: 27.8 kg/m2 in males and 26.3 kg/m2 in females. The village inhabitants are short people with a rather stocky built (mean height is 1.64 m in males and 1.50 m in females).

### Genetic Analysis: Microsatellites and SNPs

Genomic DNA was extracted from 7 ml of EDTA-treated blood with standard methods. Genotyping of all the samples collected in Talana was done in collaboration the NHLBI Mammalian genotyping Service of the Marshfield laboratory under the direction of Dr. James L. Weber. Six hundred and fifty-four markers distributed over the genome were genotyped. To obtain a higher average marker density, we utilized approximately an additional 400 markers selected from the ABI PRISM^® ^Linkage Mapping Set version 2.5 HD5 (Applied Biosystems, Foster city, CA). Polymerase chain reaction was carried out according to standard protocols. Microsatellite products were loaded on ABI PRISM 3730 DNA Analyzer (Applied Biosystems) and data were processed by GENEMAPPER v3.0 software. A total of 902 highly informative (mean heterozigosity = 0.7) microsatellites distributed over the genome (average spacing = 4.3 cM), except X and Y chromosomes, were used for the statistical analysis.

SNPs genotyping was performed on the Affymetrix GeneChip platform. We used the GeneChip^® ^Human Mapping 500 K Array Set that comprises two arrays (the Nsp and Sty arrays) capable of genotyping ~262,000 and ~238,000 SNPs, respectively. We followed the recommended protocol described in the Affymetrix manual. All DNA samples were normalized to 50 ng/μl. Then, 5 μl (250 ng) of dsDNA was digested with the appropriate restriction enzyme and ligated to adapters using T4 DNA ligase. Samples were then PCR amplified using TITANIUM Taq polymerase on a GeneAmp^® ^PCR System 9700 gold plate thermal cycler. PCR products were purified using the Clontech purification kit followed by fragmentation. Samples were then injected into cartridges, hybridized, washed and stained. Mapping array images were obtained using the GeneChip Scanner 3000 7 G plus. Genotypes were called using the BRLMM (Bayesian Robust Linear Model with Mahalanobis distance classifier) software. Individual arrays not passing the 93% call rate, threshold at P = 0.33, were considered a quality control failure and subsequently re-genotyped.

The data (microsatellites and SNPs) were checked for typing inconsistencies with the pedigree structure using PedCheck [23], employing semi-automatic procedures to investigate and to remove errors. Instances that could not be resolved were treated as missing data.

After the above-mentioned data cleaning, additional genotypes that were determined as erroneous by Merlin default error-detection option, were removed.

Recently, we performed a population genetic study on Ogliastra villages, including Talana, in order to compared the extent of LD using a set comprising both commons and informative SNPs in all communities and in a standard outbred population (30 HapMap CEU trios) [19]. The mean heterozygosity of the markers was 0.345 and the average of minor allele frequency (MAF) was 0.251. The monomorphic SNPs (M-SNPs), were ~26% of the entire SNPs list. The comparison with the HapMap samples showed that 88.5% of the shared M-SNPs were monomorphic also in the CEU population and 10.89% showed a MAF > 0 and <0.05. The metric LD map evidence that the pattern of "plateaus", regions of high LD, and "steps", regions of increased recombination tends to be shared among Ogliastra vs CEU population. This pattern presumably reflects the common distribution of recombination hot spots across various populations. With the intention of using SNPs genotypes for linkage analysis, we create a informative set of SNPs useful for linkage studies using only the informative list of the SNPs in both populations.

We selected a subset of 16300 highly informative biallelic markers: we used as a cut off a Minimum Allele Frequency (MAF) >0.02 and average spacing of 150 kb in reason of the best compromise among informativity, spacing and number of SNPs. All markers physical coordinates refer to the NCBI September 2006 released annotation update for the human genome (NCBI Build 36.2). Alleles are expressed in the forward (+) strand of reference.

### Families' selection

**Figure 1:**
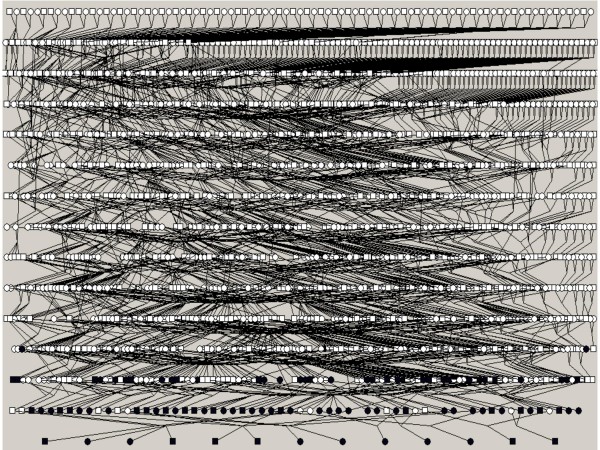
**The entire pedigree with 2,246 members, including 99 hypertensive patients, from the Talana population**. Men are represented with squares and women with circles. Affected individuals are represented in black. To simplify, unaffected relatives of the patients included in the analyses are not shown. This pedigree was drawn using Pedfiddler v. 0.5.

We reconstructed a single deep-rooted 16-generation pedigree that includes all the population of the Talana village encompassing 5219 individuals with multiple inbreeding loops. Mean inbreeding calculated from the genealogy using a subset of 876 living individuals is 0.018 [SD ± 0.022]. All 99 selected affected individuals were clustered in a smaller pedigree including 2,246 people in order to reduce the number of subjects that was not directly connected to the samples used in the present study (see figure 1). The mean kinship of 99 affected individuals was 0.0229. The complexity of these large genealogies, typical of isolated populations, forbids using them directly for linkage analysis and forces to break-up the pedigree in sub-branches.

The clustering of affected individuals in various separate families was performed using a semiautomatic approach performed by a particular tool called FamPrep implemented in the PedNavigator software [21]. This software, starting from a list of subjects, allows to select the number of generations and subsequently reconstructs various genealogies. Selecting a mean number of 4 generations we obtained 16 families that include 267 individuals (84 founders, 183 non-founders) comprehending 62 hypertensive subjects (mean 3.87 for family, range 2–6). The mean family size was 16.68 samples (range 8–25). Of the 267 individuals 141 were genotyped with STRs and 139 with SNPs with mean frequency of genotyped individuals equal to 52.80% and 52.05% respectively. All affected individuals were genotyped with both markers sets.

### Statistical Analysis

As the inheritance pattern of EH is unknown, we used non-parametric linkage analysis to assess evidence for linkage. This type of test does not require specification of inheritance pattern, disease allele frequency and penetrance rate. Moreover it tolerates phenotype definition errors.

We used MERLIN (Multipoint Engine for Rapid Likelihood Inference) software [24] to perform multipoint NPL analysis using both the selected 16300 SNPs and 902 STRs in the 16 families' data sets. MERLIN implements the Whittemore and Halpern [25] algorithm to test for allele sharing among affected individuals and also calculates a nonparametric LOD score by use of the Kong and Cox [26] linear model.

Linkage analysis assumes linkage equilibrium between markers, but when dense maps are used, this assumption is not respected and it can lead to inaccurate results, especially when parental genotypes are missing. We corrected marker-marker linkage disequilibrium using the methods implemented by MERLIN. This algorithm clusters tightly linked markers and uses haplotypes frequencies to model LD within each cluster. Carrying out STRs analysis, we used the distance k as standard that inserts a cluster breakpoint between markers that are less than k cM apart. We used k = 2 cM. To evaluate LD values using SNPs, we created clusters using r^2^>0.4 as parameter.

The Talana-specific STRs genetic maps were constructed for all autosomal chromosomes using CRI-MAP [27] that was run on an extended pedigree where the number of informative meioses were maximized (up to 900 informative meioses). Allele frequencies were estimated from the whole sample of genotyped subjects and average markers heterozygosity was 0.71 (SD = 0.09). A confirmatory analysis was executed using Marshfield and Decode genetic map distances in the mapping analysis.

**Figure 2:**
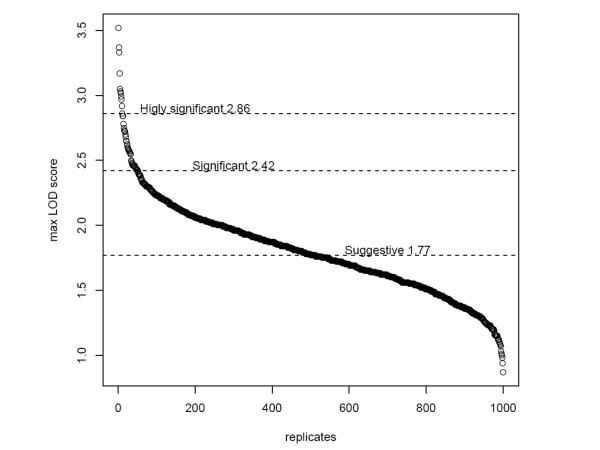
**Distribution of genome-wide maximum simulated LOD scores in each of 1,000 replicate using the STRs markers**.

Using STRs set, we evaluate genome-wide type I error rates and estimate the threshold for statistical significance by use of simulations [28]. We used the pedigree of 2,246 individuals including the 99 affected individuals and all of the 267 persons belonging to 16 families for marker simulations using the program Genedrop from the MORGAN package . Number of markers, inter-marker distances and marker allele frequencies were simulated according to the typed marker set. We performed linkage analysis using Merlin in the 16 sub-pedigrees setting to "missing" the genotypes of individuals not sampled. For each genome screen the highest LOD score was stored. Cumulative density function of the obtained maximum LOD scores approximates the distribution of the type I error rates. We performed 1000 simulations for STRs map and the results showed that LOD score of 2.42 corresponds to a genome-wide type I error of 5% and that a LOD score of 1.77 corresponds a genome-wide type I error of 50% (Figure 2). In SNPs analyses, we decided to use the standard thresholds of significance (LOD score 3.3 for significant and LOD score of 1.9 for suggestive) [29] because of high computational complexity and time consuming. Finally, we calculated the information content (IC) that represents the major advantage of using high density SNPs versus microsatellites. IC was calculated using MERLIN to compare both sets of markers in order to investigate factors contributing to different results between the two scans.

## Results

### Descriptive clinical characteristics

In Talana, we were able to collect 654 voluntary participants older than 18 years with a mean age of 52 ± 18 (see table 1). The sample included 41% males and 59% females. Within the adult population, the prevalence of hypertension was about 26% with a growing trend up to age 69, with men showing a higher age-specific rate of hypertension than women. As previously reported in national and international literature, we confirmed a direct relationship between body mass index and blood pressure levels in our populations [30,31]. BP was higher in ex-smokers than in non smokers or current smokers, respectively; it was lower in people doing sports; it increased with increasing LDL, fasting glucose, uricaemia and potassium; and it was lower in women taking oral contraceptives. Mean SBP for succeeding age decade intervals shows a tendency to rise progressively in both men and women, but mean values are always lower in women than in men. After age 60 the rate of increase becomes higher in women even if men always show higher SBP. For both men and women average DBP increases from the second to the fifth decade of age and tends to slightly decrease afterward. Overall 70% of the hypertensive population was aware of their condition and 65% were on anti-hypertensive therapy. Only 41% of the studied subjects currently undergoing treatment for hypertension exhibited blood pressure within normal limits. Hypertensive females are generally more aware, better treated and better controlled than males. Most patients under treatment take one drug. The most represented pharmaceutical classes were agents acting on the Renin-Angiotensin System, followed by beta-blockers and by calcium channel blockers.

**Table 1:** Clinical characteristics of the studied population.

	**DBP^§^** **(mm Hg)**	**SBP^§§^** **(mm Hg)**	**Age^#^** **(Years)**	**Height** **(cm)**	**Weight** **(Kg)**	**BMI^##^** **(Kg/m2)**	Tot Chol * (mg/dL)	**HDL** **(mg/dL)**	**LDL** **(mg/dL)**	**Triglyce** **rides** **(mg/dL)**	Treatment **
Males (25)	92.81 ± 8.12	146.60 ± 13.50	56.92 ± 17.50	163.25 ± 5.26	78.20 ± 8.07	29.38 ± 3.06	196.50 ± 38.53	55.10 ± 9.84	116.52 ± 31.48	124.40 ± 37.58	69.23%
Females (37)	88.91 ± 10.17	151.38 ± 15.22	58.38 ± 13.05	150.13 ± 5.16	63.83 ± 9.80	28.34 ± 4.17	206.42 ± 29.74	60.5 ± 13.55	122.71 ± 29.29	116.02 ± 49.06	59.52%
**AFFECTED** (62)	90.38 ± 9.58	149.59 ± 14.68	57.86 ± 14.73	155.05 ± 8.22	69.22 ± 11.51	28.73 ± 3.80	203.00 ± 33.045	58.63 ± 12.57	120.57 ± 29.93	118.91 ± 45.27	63.23%

Males (33)	80.56 ± 9.14	137.97 ± 15.38	63.94 ± 17.28	160.76 ± 6.20	69.73 ± 11.47	26.92 ± 3.84	190.05 ± 45.89	46.94 ± 11.55	122.49 ± 39.31	103.11 ± 41.48	-
Females (46)	80.45 ± 6.99	131.95 ± 15.64	56.35 ± 14.70	149.15 ± 5.48	57.70 ± 11.17	25.74 ± 4.95	203.79 ± 33.62	64.55 ± 14.31	120.13 ± 31.72	95.51 ± 38.59	-
**UNAFFECTED** (79)	80.51 ± 8.05	134.91 ± 15.68	60.03 ± 16.30	154.49 ± 8.21	63.24 ± 12.34	26.33 ± 4.99	199.90 ± 37.61	59.56 ± 15.68	120.80 ± 33.71	97.66 ± 39.23	-

**TALANA** (654)***	79.87 ± 13.07	131.28 ± 21.01	51.87 ± 18.52	155.78 ± 9.56	65.60 ± 14.46	26.95 ± 5.03	194.31 ± 34.45	58.39 ± 12.67	114.53 ± 29.81	106.92 ± 58.51	-

All columns contain Mean values and Standard Deviations for each variable.

§DBP, Diastolic Blood Pressure; §S SBP, Systolic Blood Pressure; # Age, Age at examination; ## BMI, Body Mass Index; * Tot Chol, Total Cholesterol, ** Treatment, % taking antihypertensive medications for at least one year; *** Analyzed subjects >18 years.

### Genetic analysis

In order to analyze our samples we started out breaking up the Talana EH pedigree creating 16 sub-pedigrees that include 62 affected subjects and 79 normal controls with an average 52% genotyped subjects in each pedigree. The phenotype characteristics of the 141 individuals used in the genetic analysis are summarized in table 1.

Using MERLIN, we performed two genome wide search (GWS): the first used 902 uniformly spaced microsatellites while the second used about 16,300 SNPs from the GeneChip^® ^Human Mapping 500 K Array Set, selected on the basis of spacing (>150 kb) and informativeness (MAF > 0.02) in the Talana population. The mean information content (IC) across 22 autosomal chromosomes for the full SNP set was uniformly higher than that for the microsatellites. The mean genome-wide IC, over all chromosomes, was 0.65 (Min: 0.60; Max: 0.68; SD: 0.027) for the microsatellites while for the SNPs was 0.716 (Min: 0.70; Max: 0.73; SD:0.009).

**Figure 3:**
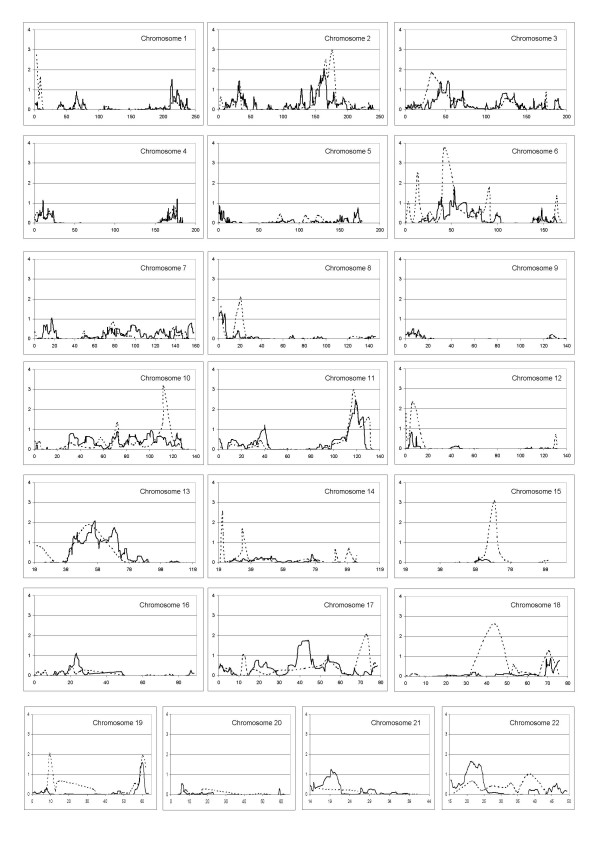
**Multipoint LOD scores for all autosomal chromosomes**. Each panel shows the chromosome number with the length in megabases (Mb) in abscissa and the LOD scores in ordinate (microsatellites-dotted line; SNPs-solid line).

**Figure 4:**
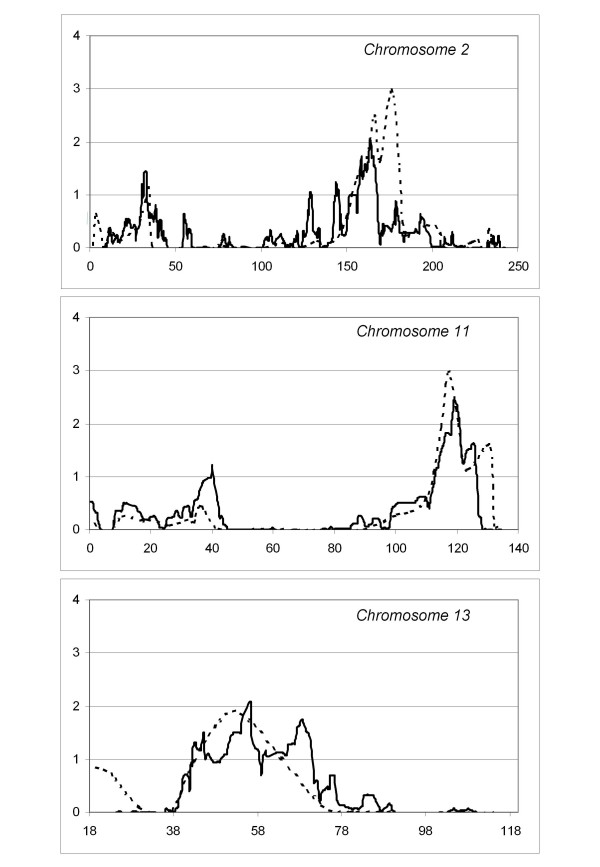
**Multipoint LOD scores for each chromosome that showed the most significant results**. Each panel shows the chromosome number with the length in megabases (Mb) in abscissa and the LOD scores in ordinate (microsatellites-dotted line; SNPs-solid line).

A total of nine regions showed significant linkage for the STRs analysis (LOD SCORES>2.42) and eight suggestive loci (LOD SCORES>1.77) were founded, while in the SNPs analysis, the standard threshold of significance do not allow us to identify significant chromosomic regions (LOD SCORES > 3.3), but only three suggestive loci (LOD scores> 1.9) (see Table 2 and Figure 3). Results of the model-free linkage analysis have evidenced three loci in both STR and SNP scans and linkage results show a remarkable degree of similarity (Figures 4) on chromosomes 2q24, 11q23.1–25 and 13q14.11–21.33. In addition, we found concordant results on three locations through both GWS: 2p23–22, 3p24.1–14.3 and 19q13.41–13.42.

**Table 2:** Summary of results in both genome wide scans using 902 autosomal microsatellites and 16.300 biallelic markers.

***Chromosome***	***Microsatellites***			***SNPs***		
	*Peak marker*	*Position (Mb)*	*LOD score*	*Peak marker*	*Position (Mb)*	*LOD score*

**1p36.32–36.21**	D1S468	3.6–9.5	2.75 *			
**2q24**	D2S2981	155.7–180.8	2.96 *	rs9287822	157.08–167.62	**2.03**
**3p24.1–14.3**	D3S2432	27.9–49.4	**1.86**	rs10490772	43.07–54.91	1.45
**6p24.1–23**	D6S1006	12.96–14.06	2.50 *			
**6p21.1–12.3**	D6S1017	41.7–50.7	3.75 *	rs4715134	48.77–50.04	1.12
**6q15**	D6S462	90.98	**1.78**			
**8p22–21.3**	D8S560	17.9–21.6	**2.06**			
**10q25–26**	D10S597	111.2–119.1	3.18 *			
**11q23.1–25**	D11S1998	114.7–123.1	2.96 *	rs7949154	112.8–126.6	**2.45**
**12p13.33–13.1**	D12S374	3.5–13.5	**2.30**			
**13q14.11–21.33**	D13S1807	41.5–54.0	**1.86**	rs7333137	44.9–70.4	**2.09**
**14q11.2**	D14S742	19.9–21.7	2.51 *			
**15q22–24**	D15S131	64.3–72	3.80*			
**17q25.1–25.3**	D17S2195	70.2–73.4	**2.01**			
**18q21.1**	D18S970	33.1–51.5	2.62 *			
**19p13.2–13.12**	D19S586	8–12.5	**2.05**			
**19q13.41–13.42**	D19S418	58.4–62.3	**1.96**	rs11672983	58.89–61.11	1.6

Significant values are in boldface with asterisk (*), suggestive in boldface.

Starting from chromosome 2, STR analysis achieved a significant maximum LOD score of 2.96 at position 175.8 Mb on 2q24, spanning a region from 155.7 to 180.8 Mb. The SNP scan confirmed and refined this locus to a 10 Mb region (157.1–167.6 Mb). SNPs maximum LOD score was 2.03 at position 163.5 Mb.

Both GWS show a similar result (STR max LOD = 2.96; SNP max LOD = 2.45) on chromosome 11q23.1–25. Microsatellite analysis identified a significant locus from 114.7 to 123.1 Mb with a max peak for D11S1998 (117.2 Mb). SNPs analysis detected a region from 112.8 to 126.6 Mb with maximum LOD of 2.45 at 119 Mb. The inspection of peaks position obtained with the 2 analyses reveals that they are in a region where microsatellites coverage and information content is lower than average compared to the rest of the chromosome. The mean information content for SNPs was 0.70 (range 0.63–0.75) and for microsatellites was 0.65 (range 0.55–0.68). This led us to consider the SNPs localization as more precise.

Our double analysis identified an extensive region on chromosome 13q14.11–21.33. SNPs analysis showed a locus spanning about 30 Mb (44.9–70.4 Mb) with max LOD score of 2.09 at 56.3 Mb. The region identified with STRs is smaller (41.5–54 Mb, peak marker D13S1807, LOD score 1.86) but it is not well defined because there are no markers covering the region between 60 and 74 Mb.

On chromosome 19q13.41–13.42 STRs and SNPs analyses show a perfect overlap in a region of about 4 Mb in size (58.4–62.3 Mb) but the results showed only a suggestive threshold for STRs analysis. This result is strengthened by informative sets of markers for both analyses: at 60.2 Mb with STRs analysis (LOD score 1.96) and at 60 Mb with SNPs analysis (LOD score 1.60).

For chromosome 3p24.1–14.3, the results we obtained were suggestive for STRs analysis, given the overlap and pattern similarity but the statistical significance was lower what obtained for other loci described in this article. Therefore we considered the microsatellites IC not satisfactory for the entire region.

Similar results were obtained on chromosome 2 where linkage was confirmed both with microsatellites and SNPs with similar patterns but where the peak values were low (D2S367 = 1.14 and rs2710605 = 1.44).

The microsatellite NPL analysis found several additional loci with significant LOD SCORE>2.42 not confirmed by the biallelic variants analysis. The microsatellites analysis identifies significant results on the following regions: 1p36.32–36.21, 6p24.1–12.3, 10q25–26, 14q11.2, 15q22–24 and 18q21.1 (see table 2) and suggestive results on 6q15, 8p22–21.3, 12p13.33–13.1, 17q25.1–25.3 and 19p13.2–13.12. The large part of them were isolated peaks on a single marker surrounded by negative values except for the 6p24.1–12.3 locus that partially overlap to SNPs results with very low LOD score values (rs4715134 = 1.12). There are several possible explanations for these discordant results such as maps coverage and differences in IC.

## Discussion

We performed Genome-wide Search using two different systems, STRs and SNPs, with the aim of identifying and confirming susceptibility loci associated to essential hypertension by comparing the results obtained. We conducted the analyses on samples obtained in an isolated founder population (the village of Talana) in which genetic heterogeneity and environmental noise are likely to be reduced.

We carried out a careful epidemiological investigation of Talana's population in order to assess the environmental and clinical features of the selected object of our study. This section of the study is important because there are very few epidemiological studies in particular in Sardinia where we have limited epidemiological data coming primarily from the main hospitals in Cagliari (the main city of the island). The first phase of the study had the purpose of determining the prevalence of common diseases and quantitative traits presenting familial aggregation. One of the aims was to verify if the essential hypertension clinical and epidemiological features found in this peculiar isolate are comparable with those observed in large out-breeding populations. Prevalence, determinants, awareness and treatments of hypertension were comparable and similar in trend to those reported in large epidemiological surveys. We found that the prevalence of hypertension in the village was about 26%, pretty close to the 27% prevalence worldwide. Hypertension prevalence for Talana was lower than that published by the Italian Istituto Superiore di Sanità for Sardinia as a whole they report a prevalence of EH of 29% in females and 33% in males considering as hypertensive a subject with a SBP/DBP > 160/95 mm Hg or under pharmacological treatment, while for Italy as a whole, they report a prevalence of hypertension of 31% in females and 33% in males. Although we used less restrictive criteria we found a lower prevalence of EH in these isolated villages. This result could be due to the substantially rural nature of these communities, characterized by a healthier environment, nutrition and life style. Similar conclusions have been suggested in studies carried out in several countries, where it has been found that the mean blood pressures of subjects living in transitional and urban areas were significantly higher than those of subjects in rural areas [32,33]. Ogliastra estimates are comparable to those described in non Hispanic whites and Mexican Americans and they are lower than those described in the other countries.

We performed a non parametric linkage analysis, the most common approach used when, as in the case of EH, it is not possible to define a clear transmission pattern within families. To gain in power in order to detect linkage we performed two genomewide search, the first using microsatellites and the second using SNPs to benefit from a more dense map and from higher IC [34]. We decide to use four generation families in reason of the minimal loss of IC. Sub pedigrees connecting more distantly related affected individuals tend to include more non-genotyped ancestors/individuals, which may lead to smaller ICs and therefore decrease power [35]. We performed additional analyses using different sets of families, starting from the same cohort of patients, created by different software (GREFFA, PedCut) and using different linkage software (data not shown), and we confirmed the same loci with the same peak markers of the analysis described in the results section.

We identified several loci associated to EH. We considered particularly relevant the loci with significant e/o suggestive LOD scores obtained both with SNPs and STRs scans. Further support to consideration of positive loci came from their previous association to hypertension or to cardiovascular disease phenotypes in the published literature.

The potential importance of the identified locus on chromosome 2q24 in the control of blood pressure is supported by previous genome-wide scans and association studies in other human populations. Specifically, 5 genome-wide linkage screens detected a signal overlapping with our region and we can make a direct comparison of the chromosome 2q locus identified in Talana families with the other studies. Zhu et al, [36] found linkage to chromosome 2q in Chinese hypertensive sibling pairs and found a linkage peak around marker D2S142. The same marker represent also 1 of 4 loci associated with hypertension in the BRIGHT study performed in 1599 severely hypertensive British families [37]. Two independent GWS on Finnish population found suggestive evidence of linkage for hypertension on chromosome 2q: the first identified a 17-cM region from 169.4 to 186.8 cM [38] and the second split the region suggesting 2 susceptibility loci with a peak (LOD = 1.8), adjacent to our candidate region. Recently, in a Kyrgyz family in which hypertension appears at an early age and where several family members have suffered stroke, genome-wide linkage screen and critical recombination events observed within this family suggest that an hypertension associated locus may lie within a single <10-cM area of chromosome 2q24.3 to q31.1 between markers D2S2380 and D2S335 [39] with a LOD score of 2.67 for marker D2S2330. Moreover, in hypertensive rat models a blood-pressure quantitative trait locus located on rat chromosome 9q22-q34 exhibits high homology with our region of interest on 2q.

Furthermore, we have recently published a linkage study, that identified a locus for total serum cholesterol and LDL cholesterol on chromosome 2q21.2-q24.1 [40]. In light of the high BMI values in our population, we can suppose the presence of a common variant underlying HTN and cholesterol confirming a possible direct relationship between BMI, lipid profiles and blood pressure levels in our populations.

Our SNPs analysis restricted the candidate region to the first part of the identified region. The current release of the human genome assembly, indicates that the 2q24.3 to q31.1 region between markers rs526540 and rs3914147 (157.081 to 167.624 Mb) encompasses 70 genes of known or predicted function. None of the genes responsible for the monogenic forms of hypertension are found within this region. However, we have identified 5 well-characterized sodium channels, voltage-gated alpha subunit genes (SCN1A, SCN2A, SCN3A, SCN7A and SCN9A) that could be considered biologically plausible candidates. Particularly, SCN7A codes for the a-subunit of a cardiac and skeletal muscle sodium channel protein. A polymorphism in exon 18 of this gene was associated with essential hypertension among the Chinese Han population of Shanghai, China [41]. Recently the major allele T of the cv356952 biallelic marker was significantly associated with higher left ventricular mass index in a cohort of hypertensive black siblings [42].

Furthermore, 4 biallelic markers with the highest LOD score were placed on the potassium voltage-gated channel, subfamily H (eag-related), member 7 gene (KCNH7). Two markers are located on the gene (rs9287822, rs4667468) and the others lie in the strong linkage disequilibrium section of the 3' region of the gene (rs11900934, rs16848123). The voltage-gated potassium channels represent the most complex class of voltage-gated ion channels from both functional and structural standpoints. Their diverse functions include regulating neurotransmitter release, heart rate, insulin secretion, neuronal excitability, epithelial electrolyte transport, smooth muscle contraction, and cell volume. Murine models proved that ion channels encoded by ether-à-go-go-related genes (eag) have been implicated in re-polarization of the cardiac action potential and also as components of the resting membrane conductance in various cells [43].

We also confirmed the association of hypertension related phenotypes to the 11q region detected by 2-stage genome screen on Caucasian in United Kingdom [44]. Sib-pair linkage analysis identified a single locus on chromosome 11q on the same marker we identified with our GWS assay (D11S925). In addition, the same locus was replicated in the HERITAGE Family Study on black individuals [45]. In this genomic region, close to the highest peak we identified two genes encoding for sodium channels (SCN4B and SCN2B). Four different β-subunits have been described and all are detectable in cardiac tissue. Recently, a missense mutation (L179F) of the SCN4B gene was associated to heritable arrhythmia syndrome throughout a 3-generation pedigree and it is surmised that it may contribute to the alteration of the cardiac sodium channel current [46].

Regarding the 13q14.11–21.33 locus, the genomic region identified in our study is not well enough defined to justify a gene search since both STRs markers and SNPs analyses do not consent to focus on a restricted area. But also in this case, we find the same location associated to hypertension in meta-analysis and other genome scans reported in literature [47,48].

On chromosome 19 we found a STRs suggestive region with a precise overlap between SNPs and STRs analysis in a region of only 4 Mb in size. As previously, we replicated a suggestive result obtained on a cohort of 1174 individuals (562 males and 612 females) that were classified as having hypertension from the Framingham Heart Study. This genomic interval includes several potential candidate genes and in particular a cluster of three gamma subunit genes: CACNG6, CACNG7 and CACNG8.

Finally, particular attention was focused on the first part of chromosome 2 where, in a previous study, we identified an hypertension associated locus carrying out GWS on a single Talana family [49]. In that study, we utilized a less dense map of markers, about 400 microsatellites, and we selected only the most severely hypertensive subjects (diastolic blood pressure (DBP) >100 mm Hg) belonging to a single deep-rooted pedigree (12 generations). The present analysis confirmed the signal both with microsatellites and SNPs, the 2 different GWS presenting similar patterns, on the 2p22–23 region shifting the previous signal of about 8 Mb but the peak values were low (D2S367 = 1.14 and rs2710605 = 1.44). The pattern of our results using STRs and SNPs have a high similarity with the signals detected by Caulfield et al., [31] for the entire chromosome 2 and validate the association of this locus to hypertension. In recent times, a number of studies focused on hypertension and on QTLs influencing blood pressure confirmed this candidate locus in a region that reach over 2p24–25 to 2p22–23 in Chinese, Mexican American, African and African American, Caucasian and British people [50-56].

## Conclusion

The genome-wide screens published to date for hypertension genes are diverse in phenotype, ethnic origin, selection criteria, numbers and structures of families and range from analysis of single large pedigrees to various hundreds of sib-pairs or families. Our genome-wide scan differs from many others because to gain in power in order to detect linkage we performed two genomewide search, the first using microsatellites and the second using SNPs to benefit from a more dense map and from higher IC in the same group of families from a little village. The loci we identified in this study are clearly unlikely to reflect all the potential genetic effects on EH. However, our results prove that our population possesses genetic characteristics comparable to outbreeding population since the four loci identified in this study confirm previously identified genomic regions in association with blood pressure disease phenotypes. We are going to replicate our positive results in a large scale cohort of subjects from other villages in the same area in order to study a population with a highly homogenous genetic background as is the people from Ogliastra and it is useful to conduct further analysis to restrict the regions of interest and identify the genes involved in the etiology of EH.

## Competing interests

The research group was employed by Shardna Life Sciences while carrying out this work.

## Authors' contributions

EM, MPC contribute to study design, data analysis and drafted the manuscript. MF and NP performed part of the statistical analyses. VC, CF, IP, AS, AP and DP performed the marker analyses. MA and DS established the cohorts and provide the clinical data. GB performed the epidemiological analyses. MP provided concepts and ideas. AA provided concepts, ideas, study design, data analysis and write the manuscript. All authors have read and approved the final version of the manuscript.

## Pre-publication history

The pre-publication history for this paper can be accessed here:


